# Symbiotic Performance of Herbaceous Legumes in Tropical Cover Cropping Systems

**DOI:** 10.1100/tsw.2001.345

**Published:** 2001-11-10

**Authors:** Basil Ibewiro, Martin Onuh, Nteranya Sanginga, Bernard Vanlauwe, Roel Merckx

**Affiliations:** Laboratory of Soil Fertility and Soil Biology, Katholieke Universiteit Leuven, Heverlee, Belgium

**Keywords:** inoculation, in situ mulch, lablab, land equivalent ratio, live mulch, maize, mucuna, N_2_ fixation, Nigeria, Rhizobium, soil fertility, West Africa

## Abstract

Increasing use of herbaceous legumes such as mucuna (Mucuna pruriens var. utilis [Wright] Bruck) and lablab (Lablab purpureus [L.] Sweet) in the derived savannas of West Africa can be attributed to their potential to fix atmospheric nitrogen (N_2_). The effects of management practices on N_2_ fixation in mucuna and lablab were examined using 15N isotope dilution technique. Dry matter yield of both legumes at 12 weeks was two to five times more in in situ mulch (IM) than live mulch (LM) systems. Land Equivalent Ratios, however, showed 8 to 30% more efficient utilization of resources required for biomass production under LM than IM systems. Live mulching reduced nodule numbers in the legumes by one third compared to values in the IM systems. Similarly, nodule mass was reduced by 34 to 58% under LM compared to the IM systems. The proportion of fixed N_2_ in the legumes was 18% higher in LM than IM systems. Except for inoculated mucuna, the amounts of N fixed by both legumes were greater in IM than LM systems. Rhizobia inoculation of the legumes did not significantly increase N_2_ fixation compared to uninoculated plots. Application of N fertilizer reduced N_2_ fixed in the legumes by 36 to 51% compared to inoculated or uninoculated systems. The implications of cover cropping, N fertilization, and rhizobia inoculation on N contributions of legumes into tropical low-input systems were discussed.

## INTRODUCTION

Enhancement of soil fertility through the use of herbaceous legumes offers an attractive source of nitrogen (N) to smallholder farmers who have seriously degraded fields in the derived savanna of West Africa. Mucuna (*Mucuna pruriens* [L.] DC. var. *utilis* [Wright] Bruck) is a herbaceous legume that has received attention in West Africa for soil fertility improvement[1,2] and pest/disease control[3,4]. Lablab (*Lablab purpureus* [L.] Sweet) is an important tropical crop, although its usefulness for food, fodder, and cropping systems has not been fully developed.

Mucuna and lablab are used in both *in situ* mulch (IM) and live mulch (LM) cover cropping systems in the tropics[5]. IM involves the planting of food crops into residues of a legume cover crop usually grown solely on the same spot. LM is a crop production system in which herbaceous legume cover crops are planted directly into food crops. Some important features of LM systems may include higher and improved yield stability compared to monocrops[6], improved utilization of environmental resources[7], and reduced incidence of pests and diseases.

Interest in using legume cover cropping systems for restoring degraded soils in the tropics has been stimulated by the possibility that the legumes may fix atmospheric N and therefore not depend on soil N pool. However, some recent studies have observed that mucuna did not nodulate in all situations or nodulated ineffectively in some situations in the derived savanna of West Africa[8]. Smallholder farmers usually grow these herbaceous legumes without inoculation and fertilizer input. Thus, the $N_2$-fixing capabilities of the legumes are probably not fully reached. Few quantitative data are presently available on the $N_2$-fixing potentials of mucuna or lablab under IM or LM systems in the derived savanna of West Africa.

This study examines the effect of *Rhizobium* inoculation and urea-N fertilizer on $N_2$ fixation in mucuna and lablab under IM and LM cover cropping systems.

## MATERIALS AND METHODS

The experiment was conducted in an imperata- (*Imperata cylindrica*) infested field at Ijaiye near Ibadan, Nigeria. The field had a split-plot design with mainplot treatments consisting of rhizobia inoculation of mucuna and lablab, N fertilization (90 kg urea-N $ha^{-1}$), and uninoculated mucuna and lablab (indigenous rhizobia and not N-fertilized). Subplot treatments were: (1) mucuna (IM); (2) mucuna + maize (LM); (3) lablab (IM); (4) lablab + maize (LM); and (5) maize (IM). Ibewiro et al.[5] gave detailed descriptions of the field design and layouts. Plant spacing was 0.25 × 0.75 m for both cover crops and maize. Mucuna or lablab and maize were interseeded in alternate rows in the LM systems. Maize sole-crop systems were controls in which the vegetations were removed and imperata roots/rhizomes excavated. Plot layout was arranged in a randomized complete-block design with four replications. Each of these systems was established with a broadcast dressing of 30 kg P $ha^{-1}$ as single superphosphate and 30 kg K $ha^{-1}$ as KCl before sowing maize. N was applied only in the N-fertilized mainplot treatments, in three equal splits of 30 kg N $ha^{-1}$ each at 2, 4, and 6 weeks after sowing maize. In inoculated mainplots, the herbaceous legume seeds were inoculated with 5 ml $hole^{-1}$ of the respective broth cultures containing $10^7$ cells $ml^{-1}$ of a mixture of three effective *Rhizobium* strains isolated from the site to increase the effective rhizobia population. Ammonium sulphate ($[NH_4]_2SO_4$, 5% atom $^{15}N$ excess) was applied as solution spray on the soil surface at a rate of 10 kg N $ha^{-1}$ in the microplots at crop emergence. An equivalent rate of unlabeled $(NH_4)_2SO_4$ was applied to the remainder of the plots. Plants were harvested at 4, 8, and 12 weeks; dried in forced air at 70°C for 72 h; ground to pass through a fine mesh screen; and analyzed for total N and $^{15}N$ enrichment using a Europa Roboprep Scientific C/N Analyzer. Nitrogen fixation in the legumes was calculated using the $^{15}N$ isotope dilution technique[9] with maize as the reference plant and expressed either as proportion (%Ndfa) or amount (Ndfa) of fixed N in the legumes derived from the atmosphere.

$$\%\text{Ndfa} = 1 - \frac{\text{atom\% } ^{15}\text{N excess (legume)}}{\text{atom\% } ^{15}\text{N excess (reference crop)}} \times 100 \quad (1)$$

$$\text{Ndfa (kg N ha}^{-1}\text{)} = \frac{\%\text{Ndfa}}{100} \times \text{total N (legumes)} \quad (2)$$

The biological efficiency of dry matter production of the component herbaceous legumes under LM systems with maize measured with the Land Equivalent Ratio (LER) was calculated according to Willey[6]:

$$\text{LER} = \frac{\text{yield LM}_{\text{legume}}}{\text{yield IM}_{\text{legume}}} + \frac{\text{yield LM}_{\text{maize}}}{\text{yield IM}_{\text{maize}}} \quad (3)$$

An LER >1 indicates a better system use efficiency of the LM over IM systems in terms of environmental resource use, whereas LER value <1 indicates an IM system advantage over LM system. Data were examined by analysis of variance (ANOVA) using General Linear Model (GLM) procedures and comparisons of treatment means made by the least significant difference (LSD)[10] when there were significant treatment effects at 5% probability level.

## RESULTS AND DISCUSSION

Dry matter yield of the legumes at 12 weeks was two to five times higher in IM than LM systems (Table 1). LM with maize resulted in 42% reduction of mucuna biomass yield at 12 weeks, while it caused an 82% reduction of lablab biomass yield relative to the sole cropped legumes. Conversely, maize biomass yield in LM with mucuna at 12 weeks was reduced 12%, while lablab caused a 9% reduction of maize dry matter yield compared to biomass yield of sole cropped maize (not shown).

The LER of mucuna and lablab shoot under LM with maize at 12 weeks varied from 1.08 to 1.30. Consequently, the system use efficiency varied from 0.31 to 0.61 for mucuna and –0.02 and 0.28 for lablab (Fig. 1). Thus, 28 to 61% more land would have to be used in IM systems in order to obtain similar dry matter yield to LM systems, if each LM component crop was allocated 50% of the land. Values of LER larger than unity have often been reported when legumes and cereals are grown together[11]. The intercrop advantage of legumes and cereals is presumed to be associated mainly with the complementary use of N sources by the component legume crops[7]. N fertilization reduced the system use efficiency for mucuna shoot dry matter production by 49% and for lablab by 55% compared to the uninoculated plots. Use of resources for mucuna biomass production in the rhizobia-inoculated plots was similar to values in N-fertilized plots but less than in uninoculated treatments. Rhizobia inoculation of lablab enhanced LER compared to values in either the uninoculated or N-fertilized plots. Reduction of LER values in N-fertilized treatments in this study was in agreement with previous reports that N fertilization reduces LER of mixed crops of legumes and nonlegumes[12]. Factors that reduce $N_2$ fixation in the LM systems (such as increased concentrations of soil inorganic N) can also decrease LER. Increased concentration of soil inorganic N results in improved growth of the associated maize in the LM system, resulting in increased shading of the legumes, consequently there are reduced LER values.

N accumulation in the legume biomass at 12 weeks ranged from 49 to 421 kg N $ha^{-1}$ (Fig. 2). The amount of N accumulated by mucuna under LM was 47% less than values in the IM systems. Similarly, accumulation of N in lablab under LM systems was 84% less than total N accumulated by lablab in the IM sys-

**TABLE 1**

**Effect of Rhizobia Inoculation, N Fertilization, and Cropping Systems on Shoot Dry Matter (t DM ha$^{-1}$), Nodule Numbers (plant$^{-1}$), Fresh Weight (g plant$^{-1}$), and N2 Fixed (%Ndfa) in Mucuna and LabLab in the Field**

| Crop species | Dry Matter | | Nodule | | | | %Ndfa | |
|---|---|---|---|---|---|---|---|---|
| | *in-situ* mulch | *live*-mulch | *in-situ* mulch | | *live*-mulch | | *in-situ* mulch | *live*-mulch |
| | | | Number | Fresh weight | Number | Fresh weight | | |
| **Rhizobium inoculated** | | | | | | | | |
| mucuna | 9.2 | 5.4 | 18.2 | 1.3 | 11.2 | 0.9 | 57.0 | 93.0 |
| lablab | 11.9 | 1.7 | 11.0 | 0.7 | 6.8 | 0.2 | 64.0 | 93.0 |
| **N fertilized** | | | | | | | | |
| mucuna | 8.2 | 3.9 | 6.9 | 0.8 | 6.8 | 0.5 | 58.0 | 46.0 |
| lablab | 12.5 | 2.9 | 6.0 | 0.3 | 6.0 | 0.3 | 57.0 | 53.0 |
| **Uninoculated** | | | | | | | | |
| mucuna | 7.7 | 5.9 | 14.8 | 1.1 | 11.0 | 0.7 | 66.0 | 83.0 |
| lablab | 11.2 | 2.0 | 12.4 | 0.9 | 8.1 | 0.3 | 66.0 | 83.0 |
| $LSD_{0.05}$ | | | | | | | | |
| N source | 2.9 | 2.3 | 3.4 | 0.2 | 2.8 | 0.2 | 7.0 | 11.0 |
| species | 2.7 | 2.3 | 1.8 | 0.2 | 2.5 | 0.2 | ns[a] | ns |
| system | 1.9 | | 2.2 | 0.1 | 2.2 | 0.1 | 13.0 | |

[a]Not Significant

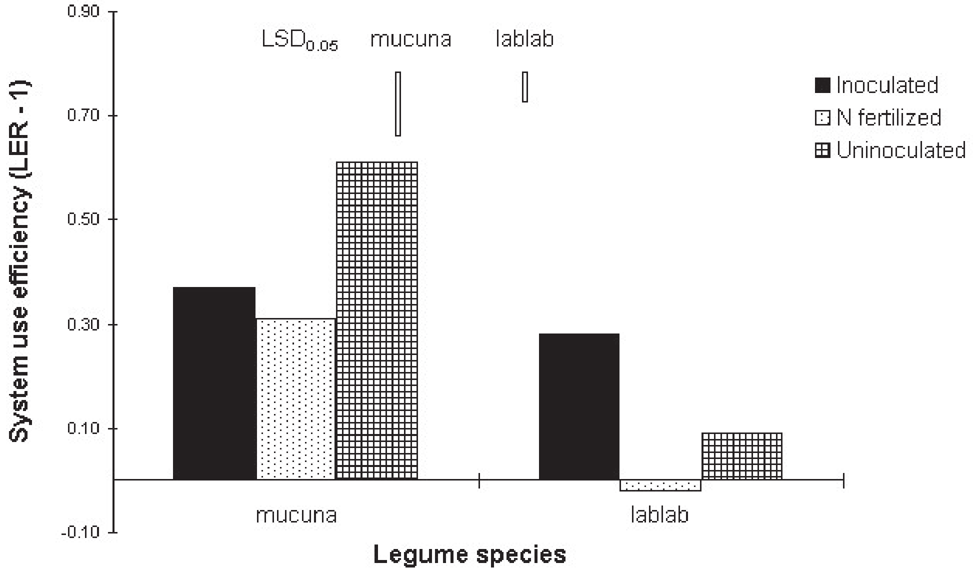

**FIGURE 1**. Effect of rhizobia inoculation and N fertilization on the systems use efficiency (LER) for dry matter production of mucuna and lablab at 12 weeks.

tems. N accumulation in maize was higher in N-fertilized treatments than rhizobia inoculated or uninoculated treatments. Nodulation of both herbaceous legumes was significantly reduced by N fertilization (Table 1). Nodule numbers and fresh weight of LM mucuna and lablab were not significantly different between inoculated and uninoculated treatments, though values tended to be higher in inoculated mucuna. LM with maize decreased nodule numbers of mucuna by 27% and by 30% for lablab compared to the IM systems. Nodule fresh weight of mucuna and lablab was reduced by 34 to 58% in LM compared to IM systems. The lack of response to introduced rhizobia strains observed in this study can be caused by competition of the introduced strain with indigenous rhizobia population. Similar results have been reported for soybeans[13]. Mucuna fixed between 54 kg N ha$^{-1}$ in the N-fertilized LM and 215 kg N ha$^{-1}$ in the uninoculated IM plots, representing 46 to 93% of its biomass N at 12 weeks (Table 1).

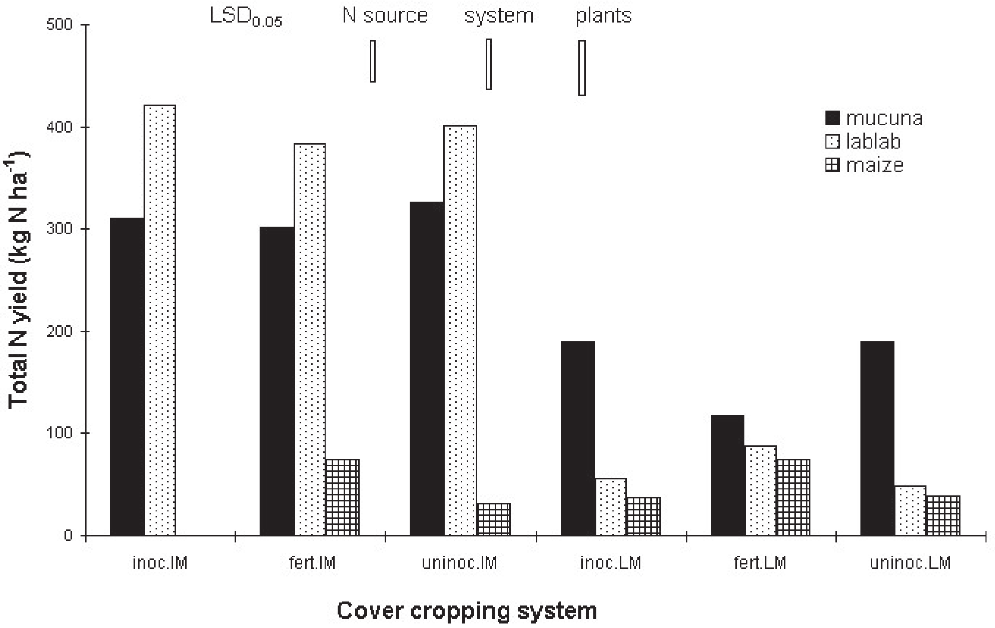

**FIGURE 2**. Effect of rhizobia inoculation, N fertilization, and cropping systems on N accumulation (kg N $ha^{-1}$) by mucuna and lablab in the field at 12 weeks.

Similarly, lablab derived 53 to 93% of its N from symbiotic $N_2$ fixation, amounting to 41 to 269 kg N $ha^{-1}$. Application of N fertilizer reduced the proportion of fixed $N_2$ (%Ndfa) in the legumes by 36 to 51% compared to the inoculated or uninoculated systems. Compared to the IM systems, %Ndfa in mucuna under LM plots at 12 weeks was increased by 14% and by 20% in lablab. Increases in %Ndfa in the herbaceous legumes under LM systems suggest that there might have been a competition for soil available N that could have stimulated N fixation in the legumes. This enhanced $N_2$ fixation could be attributed to minimized inhibitory effect of the lower available soil N concentration on $N_2$ fixation[5]. Under this condition, the legumes depend less on soil N pool. Reduced amounts of fixed $N_2$ in LM compared to the IM systems can be related to the lower dry matter yield of the legumes in LM systems, showing that biomass production should be a primary criterion to be targeted when maintaining high $N_2$ fixation by legumes in the derived savanna of West Africa. This criterion of high biomass yield should be coupled with the potential of the legumes to fix a high proportion of their N from the atmosphere.

The proportion of $N_2$ fixed in mucuna and lablab in this study ranged from 46 to 93%. These estimates of %Ndfa are comparable to those reported for other herbaceous legumes, such as *Phaseolus vulgaris*, *Pueraria phaseoloides,* and *Centrosema* spp.[8,14]. The amount of $N_2$ fixed in mucuna and lablab at 12 weeks averaged 154 kg N $ha^{-1}$. These substantial quantities of N fixed in the legumes justify their use as sources of N supply, especially in degraded cropping systems in the derived savanna zone of West and Central Africa, where it is presently not feasible or economical to introduce large inputs of mineral fertilizer.

| | | |
|---|---|---|
| Received: | July | 18, 2001 |
| Revised: | October | 27, 2001 |
| Accepted: | October | 30, 2001 |
| Published: | November | 10, 2001 |
